# Loss of Let-7 Up-Regulates EZH2 in Prostate Cancer Consistent with the Acquisition of Cancer Stem Cell Signatures That Are Attenuated by BR-DIM

**DOI:** 10.1371/journal.pone.0033729

**Published:** 2012-03-19

**Authors:** Dejuan Kong, Elisabeth Heath, Wei Chen, Michael L. Cher, Isaac Powell, Lance Heilbrun, Yiwei Li, Shadan Ali, Seema Sethi, Oudai Hassan, Clara Hwang, Nilesh Gupta, Dhananjay Chitale, Wael A. Sakr, Mani Menon, Fazlul H. Sarkar

**Affiliations:** 1 Department of Pathology, Karmanos Cancer Institute, Wayne State University School of Medicine, Detroit, Michigan, United States of America; 2 Department of Oncology, Karmanos Cancer Institute, Wayne State University School of Medicine, Detroit, Michigan, United States of America; 3 Department of Urology, Karmanos Cancer Institute, Wayne State University School of Medicine, Detroit, Michigan, United States of America; 4 Department of Oncology, Henry Ford Health System, Detroit, Michigan, United States of America; 5 Department of Pathology, Henry Ford Health System, Detroit, Michigan, United States of America; 6 Department of Urology, Henry Ford Health System, Detroit, Michigan, United States of America; National Cancer Center, Japan

## Abstract

The emergence of castrate-resistant prostate cancer (CRPC) contributes to the high mortality of patients diagnosed with prostate cancer (PCa), which in part could be attributed to the existence and the emergence of cancer stem cells (CSCs). Recent studies have shown that deregulated expression of microRNAs (miRNAs) contributes to the initiation and progression of PCa. Among several known miRNAs, let-7 family appears to play a key role in the recurrence and progression of PCa by regulating CSCs; however, the mechanism by which let-7 family contributes to PCa aggressiveness is unclear. Enhancer of Zeste homolog 2 (EZH2), a putative target of let-7 family, was demonstrated to control stem cell function. In this study, we found loss of let-7 family with corresponding over-expression of EZH2 in human PCa tissue specimens, especially in higher Gleason grade tumors. Overexpression of let-7 by transfection of let-7 precursors decreased EZH2 expression and repressed clonogenic ability and sphere-forming capacity of PCa cells, which was consistent with inhibition of EZH2 3′UTR luciferase activity. We also found that the treatment of PCa cells with BR-DIM (formulated DIM: 3,3′-diindolylmethane by Bio Response, Boulder, CO, abbreviated as BR-DIM) up-regulated let-7 and down-regulated EZH2 expression, consistent with inhibition of self-renewal and clonogenic capacity. Moreover, BR-DIM intervention in our on-going phase II clinical trial in patients prior to radical prostatectomy showed upregulation of let-7 consistent with down-regulation of EZH2 expression in PCa tissue specimens after BR-DIM intervention. These results suggest that the loss of let-7 mediated increased expression of EZH2 contributes to PCa aggressiveness, which could be attenuated by BR-DIM treatment, and thus BR-DIM is likely to have clinical impact.

## Introduction

Prostate cancer (PCa) is the second leading cause of cancer death in men in the United States killing over 32,050 men in 2010 [1]. In the past ten years, there have been significant improvements in the surgical treatment options for patients diagnosed with localized PCa. Prostate surgery has also benefited from technical and technological advancements such as nerve sparing prostatectomy and robotic prostatectomy. Adjuvant therapy, defined as additional treatment to reduce or eliminate local and distant disease, is offered to patients [2], especially those who are at high risk of recurrence (as often defined by the d'Amico criteria of PSA >20 ng/mL, Gleason 8–10, and stage T2c to T4) [3].

Radiation therapy and systemic therapy especially androgen deprivation therapy (ADT) are considered as reasonable adjuvant therapeutic options. Patients in the high-risk category have a recurrence rate of greater than 50% within their lifetime, which is unacceptable. However, one of the concerns in offering adjuvant therapy to all patients in the high risk group is that although greater than 50% of patients do recur, there are 38–50% of patients who do not [4]. To reduce the burden of over-treatment in this patient group, additional tools are needed to identify the truly high-risk patients. Current tools being studied include predictive nomograms [5] and studies evaluating gene expression profiling, which have identified a few markers of tumor aggressiveness [6], [7] although such findings have not been translated to patient management.

Since tumor recurrence and metastasis contribute to the high mortality, studies have suggested that the aggressiveness of PCa could be tightly linked with the acquisition of cancer stem cells (CSC) or cancer stem-like cells (CSLCs) characteristics. Emerging evidence suggests that deregulated expression of many microRNAs (miRNAs) including the let-7 family contributes to cancer progression and recurrence [8]. MicroRNAs are a class of non-coding RNAs of approximately 20 to 22 nucleotides in size. They regulate gene expressions post-transcriptionally by binding to a site in the 3′untranslated region (3′ UTR) of target mRNA. They have been demonstrated to regulate cell cycle, and development and progression of cancer [9]. Let-7 was first discovered and well studied in Caenorhabditis elegans. The human let-7 family consists of let-7a, let-7b, let-7c, let-7d, let-7e, let-7f, let-7g, let-7i and miR-98. The let-7 family is commonly viewed as a tumor suppressor consistent with down-regulation of oncogenes such as Ras [10], high mobility group A2 (HMGA2) [11] and c-myc [12] by binding to 3′UTR of these target mRNAs. Moreover, decreased let-7 expression was found in many cancers, including PCa [13], and it has been linked with poor patient prognosis in lung cancer [14], head and neck squamous cell carcinoma [15], and ovarian cancer [16].

Interestingly, let-7 family members have been demonstrated to regulate the self-renewal capacity of breast cancer cells [17] and PCa cells by regulating stem cell-associated factors such as Oct4, Sox2, and Nanog expression [18]. Recent studies have also documented that let-7 could regulate the expression of Lin28 and Lin28B, which in turn block the accumulation of mature let-7 [19]. This feedback regulation plays a critical role in regulating “stemness” by controlling self-renewal [20]–[23], which is the cellular characteristic of CSCs or CSLCs associated with tumor aggressiveness and recurrence. These results suggest that the let-7 family could play a key role in the progression of PCa by maintaining and regulating molecular features of CSCs or CSLCs in PCa; however, how let-7 family contributes to PCa aggressiveness is unknown.

Enhancer of Zeste homolog 2 (EZH2) is one of the targets of the let-7 family of miRNAs, and that the expression of EZH2 is strongly associated with molecular features of both normal stem cells and CSCs or CSLCs. EZH2 is a histone-lysine N-methyltransferase, a subunit of polycomb repressive complex 2 (PRC2). The polycomb group of proteins are known to be involved in the regulation of gene repression through chromatin modifications, which is essential for the maintenance of the embryonic and adult stem cells [24], [25]. PRC2 contains EZH2, Suz12, EED, and RbAp subunits and this complex is known to function in the embryonic and adult stem cells to repress developmental genes that are preferentially activated during differentiation [26]. Moreover, further studies have shown that PRC2 target genes are co-occupied by stem cell regulators such as Oct4, Sox2, and Nanog [27]. Recent studies have also suggested that polycomb group proteins are not only essential for embryonic development but also plays critical roles in tumor initiation and progression [25]. Over-expression of EZH2 has been strongly linked with cancer progression partly because PRC2 inhibits E-cadherin expression, which promotes epithelial-to-mesenchymal transition (EMT) in cancer cells [28], a phenomenon that is reminiscent of CSCs or CSLCs. Suz12 has been found to mediate E-cadherin inhibition by snail1 [29]. More importantly, EZH2 directly regulates DNA methylation by serving as a recruitment platform for DNA methyltransferase [30]. In our previous study, we found that the levels of EZH2 and Suz12 mRNA were increased in PC3 PDGF-D cells (EMT-phenotypic cells) compared to PC3 Neo cells [18]. These results clearly suggest that PRC2 can contribute to EMT characteristics of PC3 PDGF-D cells, which are consistent with signatures of CSCs or CSLCs that are associated with cancer progression and recurrence.

Previous pre-clinical studies from our laboratory have shown that indole-3-carbinol (I3C) found in cruciferous vegetables and its *in vivo* metabolite 3,3′-diindolylmethane (DIM) especially the formulated DIM (BR-DIM or B-DIM) that we have used for our phase I clinical trial [31], are potent agents in inhibiting the growth of PCa cells, which was mediated by alterations in multiple cellular signaling [32]. Our recent results showed that BR-DIM caused upregulation of miR-200b and miR-200c that are typically lost in PCa cells [33]. In the current study, we found loss of expression of let-7 family consistent with over-expression EZH2 in PCa cells and in human PCa tissue specimens, especially in tumors with higher Gleason grade. The results from correlation analysis showed that let-7 expression was inversely associated with EZH2 expression in patients with higher Gleason grade tumors. Here we also provide evidence for the role of BR-DIM (formulated DIM: 3,3′-diindolylmethane, abbreviated as either BR-DIM or B-DIM) in the regulation of let-7 and EZH2 in PCa cells as documented by our pre-clinical findings as well as findings from our on-going phase II clinical trial in PCa patients received BR-DIM prior to radical prostatectomy.

## Materials and Methods

### Cell lines and culture condition

LNCaP, C4-2B, and CWR22RV1 were maintained in RPMI 1640 (Invitrogen, Carlsbad, CA) supplemented with 10% fetal bovine serum (FBS), 10 µmol/L Hepes, 50 units/ml Penicillin and 50 µg/ml Streptomycin. Stable cell lines over-expressing PDGF-D and empty vector pcDNA3 were generated by transfection of PC3 cells with the pcDNA3-PDGF-D:His or corresponding empty vector pcDNA3 Neo as previously described and referred to as PC3 Neo, or PC3 PDGF-D cells [34], respectively throughout this manuscript. The PC3 Neo, PC3 PDGF-D, PC3, DU145 cells were cultured in RPMI 1640 medium with 2 mmol/L glutamine, 10 µmol/L Hepes (Invitrogen, Carlsbad, CA) supplemented with 5% fetal bovine serum (FBS), 50 units/ml Penicillin, and 50 µg/ml Streptomycin. PZ-HPV-7 and RWPE-1 cells were purchased from ATCC (Manassas, VA). These cells were cultured in keratinocyte serum free medium (K-SFM) provided by Invitrogen (GIBCO) and supplied with 0.05 mg/ml bovine pituitary extract (BPE) and 5 ng/ml human recombinant epidermal growth factor (EGF). All cells were maintained in a 5% CO_2_-humidified atmosphere at 37°C, and genotypically characterized to support the authenticity of these cells, which was consistent with its origin.

### Reagents and antibodies

Antibody against EZH2 was purchased from BD Biosciences (Bedford, MA). Antibody to glyceraldehyde 3-phosphate dehydrogenase (GAPDH) was purchased from Affinity BioReagents (Golden, CO). Goat anti-mouse IgG (H+L)-HRP conjugate were obtained from Bio-Rad (Reinach, BL). BR-DIM, a formulated DIM with higher bioavailability, was kindly provided by Dr. Michael Zeligs (abbreviated as BR-DIM or B-DIM; Bio Response, Boulder, CO) and was dissolved in DMSO to make 50 mmol/L stock solutions and stored at −20°C in multiple aliquots for *in vitro* study.

### Ethics statement

Patient tissues were collected after receiving the approval from Wayne State University Institutional Review Board (IRB). This clinical protocol is classified as exempt #4 categories under the NIH policy because such studies are retrospective studies using archival sample. The IRB waived the need for consent for use of the archive samples, which was consistent with NIH guidelines, and the samples were analyzed anonymously. PCa tissue specimens from our on-going clinical trial of BR-DIM (B-DIM) intervention prior to radical prostatectomy were also obtained after receiving approval from Wayne State University Institutional Review Board (IRB) and informed consents were obtained from all study subjects. Because this study is a conventional clinical trial, the IRB process required written consenting from patients prior to accrual into the clinical trial, which is routinely coordinated through the clinical trial office (CTO). The approval through IRB implicates complying with consenting and accrual without which patients cannot be enrolled into the study, and thus our study fulfilled all the required compliance guidelines from the CTO and the IRB. The local clinical trial protocol number is 2007-128, which can be found in the NIH clinical trials.gov. (http://clinicaltrials.gov/show/NCT00888654). At the time of sample acquisition for the current investigation, the study protocol accrued 20 patients; however, the study has now accrued a total of 33 patients. It is anticipated that we will close this study with 37 patients in early 2012.

### Patients and prostate tissue specimen collection

After obtaining institutional review board approval, retrospective archival pre-treatment PCa tissues and matched adjacent normal tissues were obtained from Biospecimen Core of Karmanos Cancer Institute (KCI) collected from patients who underwent radical prostatectomy from 2004–2010 at KCI. We also obtained PCa tissue specimens from our on-going clinical trial of BR-DIM (B-DIM) intervention prior to radical prostatectomy of newly diagnosed PCa patients from 2009–2011 at KCI and Henry Ford Health System (HFHS), Detroit, Michigan. Formalin-fixed paraffin-embedded (FFPE) tissues were cut for miRNA and mRNA analysis. Patients' clinical characteristics were obtained from the hospital database including race as shown in Table 1. Pathological features were ascertained from microscopic evaluation of tumor slides by pathologists both at KCI and at HFHS. Gleason score (grade) was obtained in each case from the clinical database.

**Table 1:** Characteristics of the 129 patients who underwent radical prostatectomy (RP) and the 11 patients in the BR-DIM intervention group from whom normal and tumor specimens were obtained.

Characteristics	Patients who underwent RP (N = 129)	Patients who took BR-DIM prior to surgery (N = 11)#
Age (years), median (range)	59 (45–78)	59.5 (47–64)
Gleason score		
6	44 (34.1%)	3 (27.3%)
7	52 (40.3%)	6 (55.5%)
≥8	33 (25.6%)	2 (18.2%)
PSA* (pre-intervention)		
ng/ml, median (range)	6.5 (1.1–153)	8.15 (3.2–29.14)
Race		
African American	78 (60.5%)	5 (45.5%)
European American	51 (39.5%)	4 (36.4%)
Other	0 (0%)	2 (18.1%)

* Pre-intervention PSA was available from 88 of the 129 patients who underwent RP and from 10 of the 11 patients who underwent BR-DIM intervention.

# Data for Gleason score and PSA levels were reported previously (Kong *et at.* Am J Transl Res 2012; 4(1): 14–23) and showed here for comparison with data from patients who underwent RP.

### Clonogenic assay

C4-2B, PC3 PDGF-D, and PC3 PDGF-D cells transfected with the let-7 family for 24 h were collected after trypsinization, and re-suspended in the complete medium. Single cell suspensions were plated in regular 10 cm in diameter Petri dishes at the colony density of 2,000 cells per dish. After 2–3 weeks of incubation with changing fresh media every 3–4 days, colonies were stained with crystal violet for an additional 10 min, and washed with 1× PBS. The colonies were photographed.

### Soft agar assay

C4-2B cells were harvested and suspended in culture medium. Soft agar assay was performed as previously described by our laboratory [18]. The cells were treated with 10 or 25 µM BR-DIM with changing fresh media with BR-DIM every 3 days. After three weeks of growth, the colonies were stained by incubating with 0.5 mg/ml MTT for 4 hours, and then were photographed (40×). The colony numbers were counted under a phase contrast microscope. Data were presented as colony numbers (% control).

### Self-renewal capacity assay

Single cell suspensions of C4-2B and PC3 PDGF-D cells were plated in ultra low adherent wells of 6-well plates (Corning, Lowell, MA) at 2000 cells/well in DMEM/F12 (Invitrogen) containing 1× B27 and N2 (Invitrogen). Single cell status was confirmed under microscope. Fresh medium with 10 or 25 µM was added every 3–4 days. After one week, numbers of prostaspheres were counted under a microscope and data were presented as sphere numbers per 1000 cells seeded. Prostaspheres were photographed (40×) under a phase contrast microscope.

### Western blot analysis

Total cell lysates were obtained by lysing the cells in RIPA buffer. Protein concentration was determined using BCA protein assay (Pierce, Rockford, IL). Western blotting was performed as previously described [35].

### miRNA and transfection

Cells were transfected with 40 nmol/L of let-7 precursors or miRNA precursors negative control#1 (Ambion, Austin, TX) using DharmaFECT3 transfection reagent (DHARMACON, Lafayette, CO). After 1 day of transfection, the cells were trypsonized, and re-suspended in the complete medium. Single cell suspensions were confirmed under a microscope for clonogenic assay. In addition, after 3 days of transfection, the cell lysates were prepared for Western blot analysis.

### Luciferase activity assay

PC3 PDGF-D cells with lower levels of let-7 were seeded at a density of 6×10^3^ cells/well in a 96-well plate and incubated for 24 h. The cells were co-transfected with EZH2 3′UTR luciferase plasmid (Origene, Rockville, MD) or Renilla luciferase plasmid and control miRNA, let-7a, let-7b, let-7c, and let-7d precursors using DharmaFECT duo transfection reagent (DHARMACON, Lafayette, CO). After 48 h of incubation, luciferase activity was assayed using Steady–Glo Luciferase Assay System (Promega). The Renilla luciferase activity was used as a control for transfection efficiency.

### Real-time RT-PCR

For determining mRNA levels, the total RNA from cells was isolated using the RNeasy Mini Kit (Qiagen) and the DNA was removed using an RNase-free NDase kit (Qiagen). One microgram of RNA was reverse transcribed into cDNA using a High Capacity RNA-to-cDNA Kit (Applied biosystems, Fostor, CA) according to the manufacturer's instruction. PCR was performed as previously described [18]. The relative amount of mRNA was normalized to the expression of GAPDH. For testing the miRNA levels, the total RNA from cells was isolated using the miRNeasy Mini Kit (Qiagen) and the DNA was removed using an RNase-free NDase kit (Qiagen). 20 ng of RNA were reverse transcribed into cDNA using a Universal cDNA Synthesis Kit (Exiqon, Woburn, MA) according to the manufacturer's instruction. Real time PCR was performed using specific miRNA primers (Exiqon) to quantify miRNA expression by using SYBR® Green RT-PCR Reagents (Applied biosystems). The relative amount of miRNA was normalized to the expression of RNU48. To determine the mRNA or miRNA levels in prostate cancer patient tissues, the total RNA was isolated from FFPE tissues using miRNeasy FFPE Kit (Qiagen) according to the manufacturer's instruction. RT-PCR assay for the miRNA and mRNA expression was assessed as above. The relative amount of mRNA was normalized to the expression of beta-actin and relative amount of miRNA was normalized to the expression of RNU1A.

### Microarray for miRNA profile in prostate cancer patient tissues

miRNA microarray as well as data analysis were conducted following procedures as previously described by Kong et al [18].

All data is MIAME compliant and that the raw data has been deposited in a MIAME compliant database. GEO accession number is GSE34310.

### Statistical methods

Experiments presented in the figures for cell line studies are representative of three or more independent repetitions. The data are presented as the mean and standard deviation (SD) in the bar charts. The miRNA and mRNA data from patient tissue specimen was log transformed before analysis. Comparisons of the continuous variables between two independent groups were made using the Wilcoxon rank sum test. For paired related groups (i.e. Normal tissues and paired G6 tumor tissues), the Wilcoxon signed rank test was used. Spearman correlations were used to describe the strength of linear relationship between two variables. Statistical test of significance of the correlation was based on approximated *t* distribution. All statistical tests were two sided at significance level of 0.05 unless otherwise specified. To reduce the false positivity, in other words control the family-wise Type I error, in this preliminary early phase exploratory study, we used adjusted p values from the two-stage step-up false discovery rate (FDR) controlling method [36]. A nominal error rate for estimating the number of true null hypotheses in the two-stage FDR procedure was set at 0.05.

## Results

### Expression of the let-7 family was lost in prostate cancer (PCa) tissue specimens

**Figure 1:**
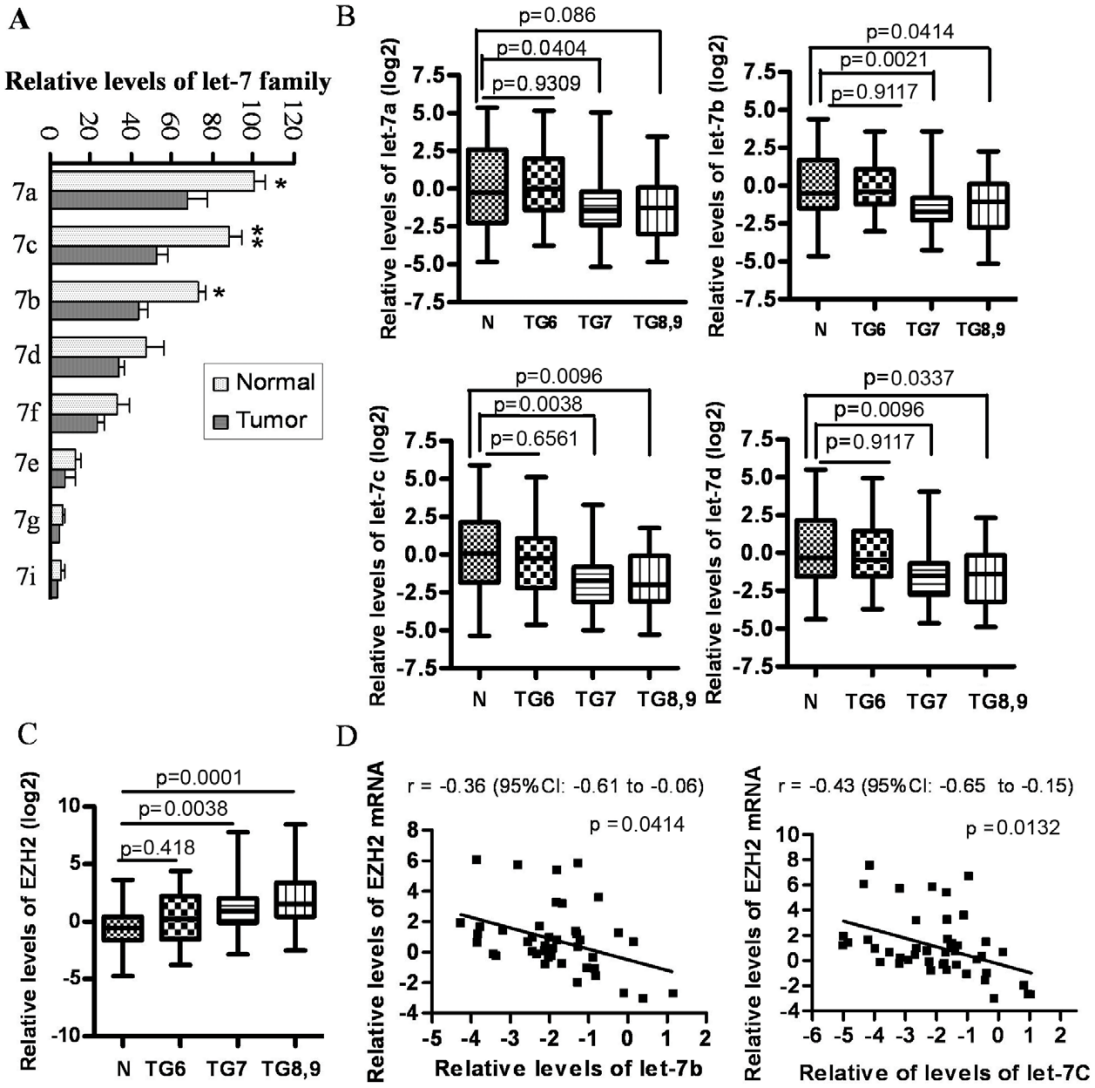
Loss of the let-7 family inversely correlated with increased expression of EZH2 in PCa tissue specimens compared to adjacent normal prostate tissues. (A) Microarray profiling was done for assessing the expression of miRNA using total RNA extracted from three PCa tissue specimens and matched adjacent normal prostate tissues. The results showed that let-7a, let-7b, let-7c and let-7d was highly expressed in prostate tissues and their expression was lost in human PCa tissue specimens (* p<0.05, ** p<0.01). (B) The results from real time-RT-PCR confirmed that the levels of let-7b, let-7c and let-7d were significantly down-regulated in tumors with higher Gleason grade. (7a: let-7a; N: normal; TG6: tumor tissues from patients with Gleason grade 6. n = 39 for normal, n = 44 for TG6, n = 52 for TG7, n = 33 for TG8, 9). (C) A significant up-regulation of the expression of EZH2 was observed in PCa tissue specimens with Gleason grade 7 (n = 46) and higher (n = 33), but not in PCa specimens with Gleason grade 6 (n = 39). (D) EZH2 levels were inversely correlated with let-7b and let-7c expressions in tumor specimens with Gleason grade 7.

To determine the levels of the let-7 family in PCa tissue specimens, we collected pre-treatment PCa tissues and matched adjacent normal tissue specimens (used as control for comparison with tumor tissues). The results from miRNA expression by mircroarray expression profiling showed that all the members of the let-7 family (miR-98 was undetectable) decreased in the PCa tissues compared to adjacent normal tissue specimens (Fig. 1A). Moreover, we found that the expression of let-7a, let-7b, let-7c and let-7d were accurately measurable compared to other family members because their expressions were very low (Fig. 1A). Therefore, we have determined and confirmed the expressions of let-7a, let-7b, let-7c, and let-7d in all the cases including 129 PCa tissue specimens and 94 adjacent normal tissue specimens. We discovered that the expression of the let-7 family in histologically normal prostate tissues from Gleason grade 7 or higher tumor was decreased compared to histologically normal tissue from Gleason grade 6 tumors (data not shown), suggesting that the histologically normal tissues from the prostate gland of patients with higher grade tumors are not normal. Thus, we took normal from the prostate of all patients diagnosed with grade 6 tumors for further analysis and used that as “normal tissue control”. We observed that the expressions of the let-7 family in grade 6 tumors are not statistically different compared to normal control. However, we found a significantly decreased expression of let-7a, let-7b, let7c and let-7d in PCa with Gleason grade 7 or higher tumors compared to normal tissue control (Fig. 1B). These results suggest that the loss of let-7 family could be associated with PCa aggressiveness especially because the lower grade tumors were no different from the normal tissue control, whereas higher grade tumors were different.

### Expression of EZH2 was increased in PCa tissue specimens and was inversely correlated with the expression of the let-7 family

Since the expression of the let-7 family was lost in PCa tissues of patients with higher Gleason grade, it raises a question of how the let-7 family could regulate PCa aggressiveness. We have searched targets of the let-7 family using TargetScan software and we found that EZH2 could be regulated by the let-7 family because there is a specific binding site in the 3′UTR of EZH2 mRNA. Thus, we have assessed the expression of EZH2 mRNA in PCa tissues. Our results showed that EZH2 expression was increased in PCa tissue specimens with Gleason grade 7 and higher compared with normal tissue control (Fig. 1C). The expression of let-7b and let-7c was inversely correlated with EZH2 expression with r = −0.36 (95% CI: −0.61 to −0.06), p = 0.0414 and r = −0.43 (95% CI: −0.65 to −0.15), p = 0.0132, respectively (Fig. 1D). These results suggest that the loss of let-7 could be responsible for increased expression of EZH2.

### Let-7 repressed EZH2 expression and inhibited clonogenic growth of PCa cells

**Figure 2:**
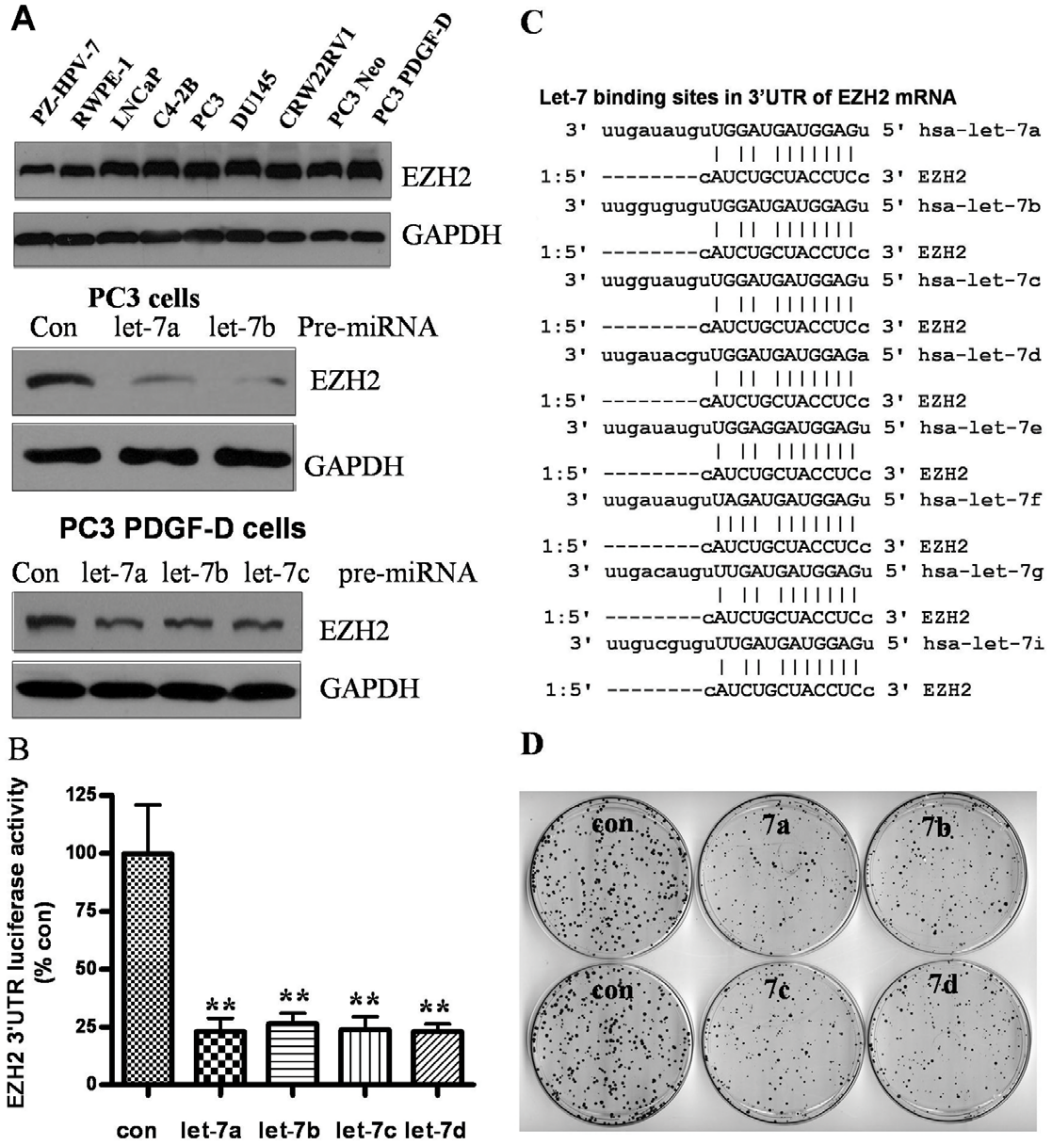
Let-7 regulated EZH2 expression, and inhibited clonogenic growth capacity of PCa cell lines. (A) Expression of EZH2 was found to be higher in PCa cell lines compared with immortalized prostate epithelial cell lines: PZ-HPV-7 and RWPE-1 (upper panel) and transfection of let-7 precursors inhibited EZH2 expression in PC3 and PC3 PDGF-D cells 3 days after transfection (middle and lower panel). (B) let-7 family members repressed EZH2 3′UTR luciferase activity in PC3 PDGF-D cells (lower levels of let-7 family in these cells) co-transfected with let-7 and EZH2 3′UTR luciferase plasmid. (C) let-7 binding sites in the 3′UTR of EZH2 mRNA were shown. (D) Transfection of let-7 precursors significantly reduced clonogenic growth capacity of PC3 PDGF-D cells (Con: control, 7a: pre-let-7a, ** p<0.01).

We examined the expression of EZH2 in PCa cell lines and immortalized prostate epithelial cell lines, and found that EZH2 was expressed in prostate cancer cells at relatively higher levels compared to immortalized prostate epithelial cell lines: PZ-HPV-7 and RWPE-1 cells (Fig. 2A, upper panel). To further determine the biological consequence of the let-7 family expression in the regulation of EZH2 expression, we transfected PC3 and PC3 PDGD-D cells with let-7 precursors, and the results showed that let-7 family members could significantly inhibit the expression of EZH2 in these two cell lines (Fig. 2A, middle and lower panel). These results suggest that let-7 family regulates the expression of EZH2. In order to gain further mechanistic insight, we tested whether let-7 could directly repress the expression of EZH2 by binding to 3′UTR of EZH2 mRNA. We co-transfected EZH2 3′UTR luciferase plasmid and let-7 precursors, and found that let-7a, let-7b, let-7c, and let-7b could strongly inhibit EZH2 3′UTR luciferase activity compared to transfection of cells with control miRNA (Fig. 2B). The let-7 binding sites in the 3′UTR of EZH2 mRNA are shown in Figure 2C. We then tested the biological consequence of cells transfected with pre-let-7 family and assessed clonogenic growth as an indirect *in vitro* measure of tumor aggressiveness. We found that the overexpression of let-7 family significantly inhibited the clonogenic growth of PC3 PDGF-D cells, which initially showed lower expression of let-7 (Fig. 2D). However, currently there is no method that could be clinically useful for the upregulation of lost miRNAs. To that end, we tested our hypothesis by testing whether BR-DIM could be useful for the upregulation of miRNAs.

### BR-DIM treatment led to the upregulation of the let-7 family and consequently down-regulated the expression of EZH2 in PCa cells

**Figure 3:**
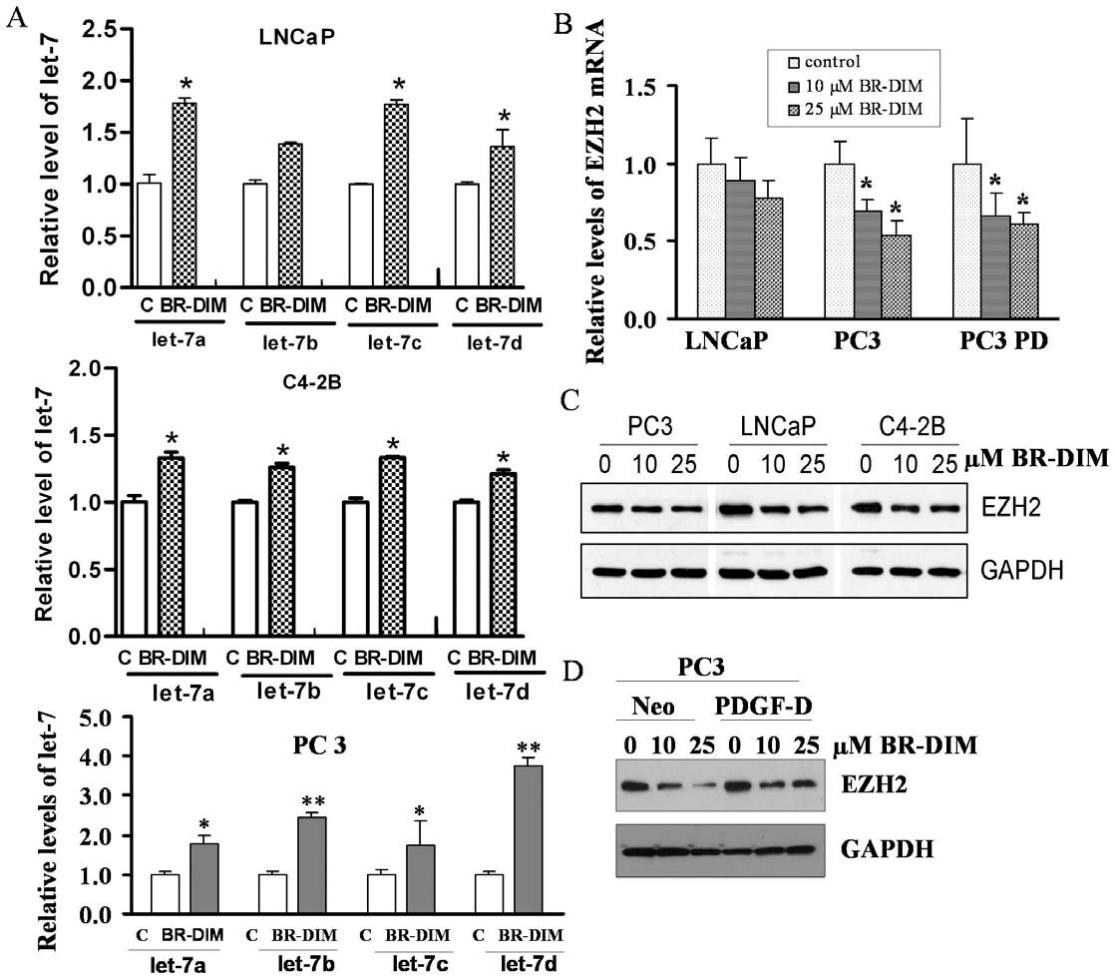
BR-DIM treatment increased let-7 and consequently reduced EZH2 expression. (A) Total RNA was isolated from LNCaP, C4-2B and PC3 cells treated with 25 µM BR-DIM for 24 h and the results from real time RT-PCR showing that the expression of let-7 was increased following BR-DIM treatment compared to untreated control (c: DMSO control). (B) Levels of EZH2 mRNA were repressed by BR-DIM treatment in a dose dependent meaner. (C and D) The cell lysates were prepared from cells treated with BR-DIM for 48 h and Western blot showing the protein levels of EZH2, which was down-regulated by BR-DIM treatment (PC3 PD: PC3 PDGF-D cells, *, p<0.05; **, p<0.01).

**Figure 4:**
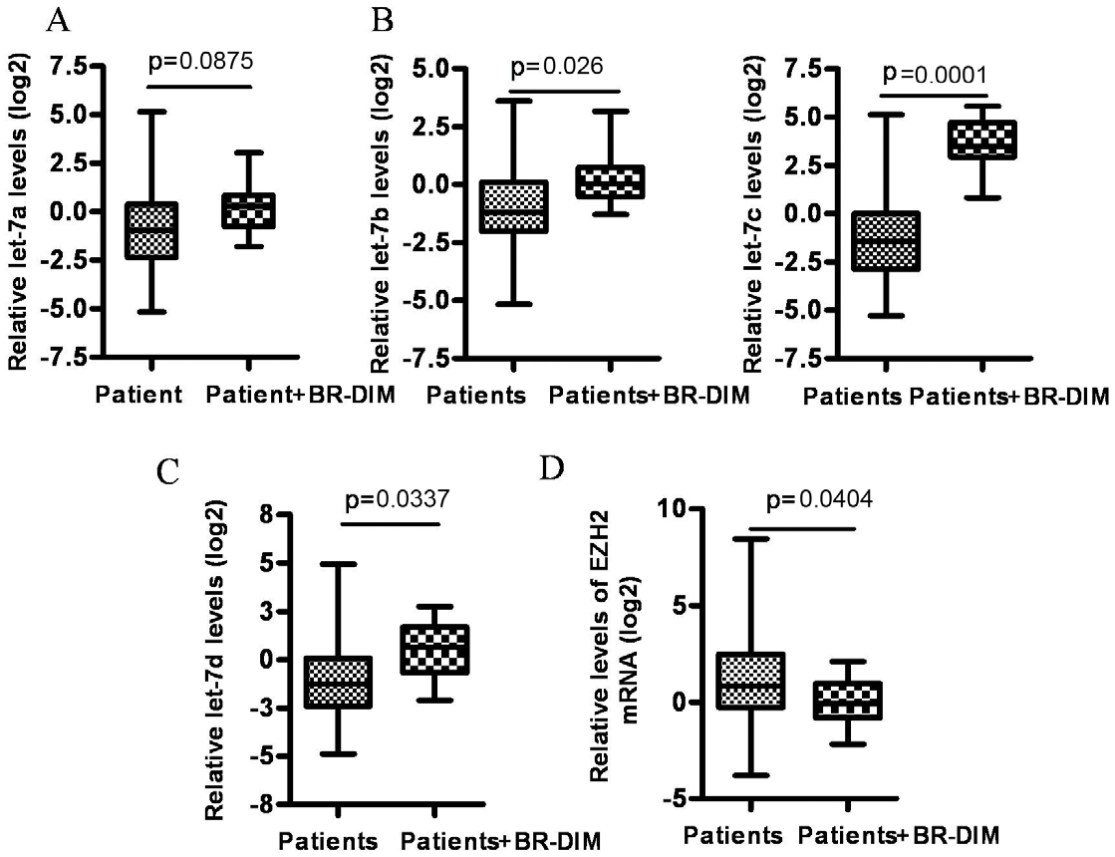
BR-DIM intervention in PCa patients resulted in the increased expression of let-7 family and consequently inhibited EZH2 expression in tumor tissues. RNA was obtained from BR-DIM intervention clinical trial samples where BR-DIM was given to patients for 2–4 weeks prior to surgery. RNA was also obtained from formalin-fixed paraffin-embedded (FFPE) tissue specimens of PCa patients with matched tumor Gleason grade, tumor stage and patient age as control group. The expression of miRNAs and mRNA was assessed using real time RT-PCR. Relative miRNA and mRNA levels were normalized to RNU1A1 and beta-actin, respectively. (A) BR-DIM intervention led to the increased trend in levels of Let-7a expression. (B and C) let-7b, let-7c and let-7d were significantly up-regulated by BR-DIM intervention. (D) EZH2 expression was down-regulated by BR-DIM intervention.

In the present study, we found that BR-DIM treatment increased the expression of let-7 family in several PCa cell lines including LNCaP, C4-2B and PC3 cells (Fig. 3A and Fig. S1A–C). Moreover, BR-DIM treatment also led to decreased expression of EZH2 mRNA (Fig. 3B) and protein in different PCa cell lines and at different time points (Fig. 3C, 3D and Fig. S1D). Moreover, More interestingly, the data from our on-going phase II clinical trial showed that BR-DIM treatment of PCa patients prior to radical prostatectomy led to the enhanced expression of let-7a, let-7b, let-7c, and let-7d in tumor specimens after BR-DIM intervention (Fig. 4A–C), and these results are consistent with decreased expression of EZH2 (Fig. 4D). Therefore, our results suggest that BR-DIM could be an important agent to re-express the lost miRNAs especially the let-7 family, which would reduce the level of EZH2 expression and compromise CSCs or CSLCs function.

### BR-DIM treatment inhibited self-renewal and clonogenic capacity of PCa cells

**Figure 5:**
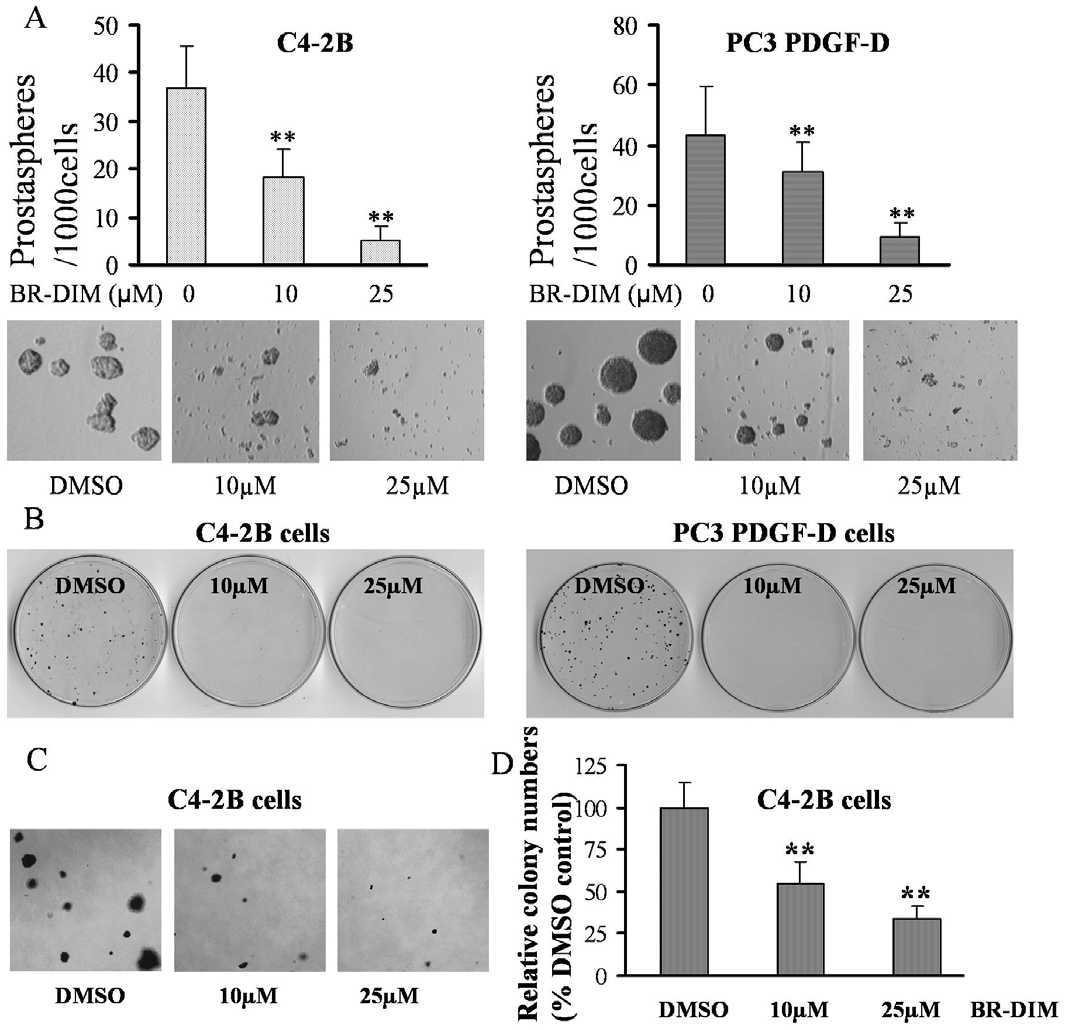
BR-DIM treatment inhibited clonogenic growth and prostasphere-forming ability. (A) Single cell suspensions of C4-2B and PC3 PDGF-D cells were plated in ultra low adherent wells of 6-well plate at 2000 cells/well in DMEM/F12 supplemented with B27 and N2 and with 10, 25 µM BR-DIM and incubated for 6 days. Prostasphere numbers were reduced by BR-DIM treatment (upper panel). Prostaspheres were photographed and the results showed that 10 and 25 µM BR-DIM significant reduced the size of prostaspheres (lower panel). (B) C4-2B and PC3 PDGF-D cells were seeded in 100 mm dishes at 2000 cells/dish, after 24 h incubation, the cells were treated with 10 or 25 µM BR-DIM for 72 h and then the culture medium was changed with fresh media without BR-DIM for every 3 days. After 2 weeks, the colonies were stained and photographed. (C and D) Soft agar assay showed that BR-DIM treatment reduced the size (left panel) and the numbers of colonies of C4-2B cells (right panel; **, p<0.01).

To test whether BR-DIM could regulate the self-renewal capacity and clonogenic ability of PCa cells, we performed sphere-forming assays and found that the treatment of C4-2B and PC3 PDGF-D cells with 10 or 25 µM BR-DIM markedly reduced the number and the size of prostaspheres compared to untreated control (DMSO control) (Fig. 5A). Moreover, BR-DIM treatment also inhibited the clonogenic growth capacity of PCa cell lines C4-2B and PC3 PDGF-D cells (Fig. 5B). The results from soft agar assay further showed that the treatment of C4-2B cells with 10 or 25 µM BR-DIM reduced the colony size and numbers (Fig. 5C and 5D), suggesting that BR-DIM could eliminate tumor cells especially the cells with CSCs or CSLCs characteristics by up-regulating let-7 family and consequently by down-regulating the expression of EZH2.

## Discussion

Although it is known that the let-7 family is associated with maintenance of stem cell signature, which is believed to be strongly linked with cancer recurrence, the mechanism by which let-7 family regulates the stem cell signatures is unknown. In this study, we found that the expression of the let-7 family was lost in PCa tissue specimens with Gleason grade 7 or higher but not in patients with Gleason grade 6 tumors. These results were consistent with corresponding increased expression of EZH2, which appears to be a target of the let-7 family. Our results suggest that the loss of expression of let-7 with a consequent over-expression of EZH2 could be associated with PCa aggressiveness. Moreover, the results from 3′UTR of the EZH2 luciferase assay and Western blot analysis further confirmed that let-7 could repress EZH2 expression by binding to 3′UTR element of EZH2 mRNA.

Recent studies have shown that miR-101 negatively regulates the expression of EZH2 [37]. Our data suggest that the let-7 family of miRNAs also be responsible for the regulation of EZH2 in human PCa. Our results showed that the let-7 family, especially let-7a, let-7b, let-7c and let-7d are highly expressed in human normal prostate tissue specimens and their expression was lost in PCa tissues, especially, in patients with aggressive (higher Gleason grade tumors) tumors. Notably, we found that the expression of let-7a, let-7b, let-7c, and let-7d was decreased in normal prostate tissues from Gleason grade 7 and higher compared with normal tissues obtained from patients with Gleason grade 6. These results suggest that the normal prostate tissues obtained from patients with higher Gleason grade tumors are not normal, which is consistent with a field-effect of prostate carcinogenesis [38].

EZH2 is a component of the PRC2, which mediates chromatin-based gene silencing through trimethylation of lysine 27 on histone H3. PRC2 also plays a critical role in the maintenance of the embryonic and adult stem cells [24], [25] as well as playing a critical role in tumor initiation and progression [26], [39]. Over-expression of EZH2 has been found to be strongly linked with the acquisition of EMT phenotype because PRC2 inhibits E-cadherin expression through trimethylation of H3 lysine 27 [28], [29], [40]. Moreover, EZH2 directly regulates DNA methylation by serving as a recruitment platform for DNA methyltransferase (DNMT) through binding of DNMTs to several EZH2-repressed genes [30], and methylation of the E-cadherin gene promoter has been demonstrated to correlate with progression of PCa [41]. DNA methylation profiles of the E-cadherin promoter were further assessed in the NCI-60 panel of cancer cells. Of the NCI-60 cancer cells, the 38 epithelial cell lines showed significantly lower (28%) methylation levels compared with the nonepithelial cell lines (58%) derived from invasive or metastatic cancer [42]. In addition, methylation mediated loss of the miR-200 family leads to E-cadherin expression as documented earlier [43], [44]. Interestingly, recent studies have shown that EZH2 inhibits the expression of the miR-200 family and miR-203 through trimethylation of H3 lysine 27 [45]. Loss of the miR-200 family and miR-203 is believed to be associated with EMT and stem cell signatures [18], [46]–[48]. Therefore, EZH2 could contribute to cancer progression and recurrence by regulating and maintaining EMT and stem cell signatures through methylation of target gene promoter and trimethylation of H3 lysine 27.

In the current study, we found that the levels of EZH2 were increased in PCa tissue specimens, especially in patients with higher Gleason grade tumors and were also highly expressed in PCa cell lines, suggesting that the over-expression of EZH2 is associated with PCa aggressiveness. Therefore, finding novel approaches by which one could re-express the lost miRNAs such as let-7 family with consequent down-regulation of EZH2 could become a newer avenue for the prevention of PCa and/or treatment of aggressive PCa. To that end, we tested the effects of BR-DIM (B-DIM), which is a “natural agent”-derived compound (formulated DIM: 3,3′-diindolylmethane by BioResponse, Boulder, CO). The results of pharmacokinetic studies of BR-DIM in mice have been reported [49] and a pharmacokinetic comparison was also reported between BR-DIM and unformulated DIM [50] showing that BR-DIM exhibits 50–60% greater bioavailability than the crystalline formulation in all tissues studied (plasma, liver, kidney, lung, heart and brain). Moreover, our phase I clinical trial results of BR-DIM in PCa patients showed that it is safe [31], which eventually led to our currently on-going phase II clinical trial in PCa patients prior to radical prostatectomy. The results obtained from eleven tumor specimens from this phase II clinical trial are exciting because it showed, for the first time, that let-7 miRNAs could be upregulated in tumors by BR-DIM intervention with consequent down-regulation of EZH2. Therefore, BR-DIM intervention could become a newer therapeutic approach since BR-DIM could cause targeted elimination of EMT phenotypic cells or CSCs (CSLCs) that contribute to tumor recurrence and metastasis as seen in patients with mCRPC. However, sample size is relatively small from our on-going clinical trial of BR-DIM intervention, and thus the effects of BR-DIM on molecular biomarkers will needs further investigation using larger number of patients to make definitive conclusion. The impact of these “proof-of-concept” findings will be further confirmed upon the completion of our on-going phase II clinical trial.

In summary, we found that BR-DIM up-regulated the expression of the let-7 family and consequently down-regulated the expression of EZH2 not only in PCa cell lines but also in human PCa tissue specimens from our on-going phase II clinical trial. These results suggest that BR-DIM could serve as a novel agent for the inhibition of PCa progression and recurrence.

## Supporting Information

Figure S1 **BR-DIM treatment upregulated let-7 expression and consequently reduced EZH2 expression in LNCaP cells at different time points.** (A–C) Total RNA was isolated from LNCaP cells treated with 25 µM BR-DIM for 8, 16 and 24 h, and the results from real time RT-PCR were shown to document the expression of let-7, which was increased following BR-DIM treatment compared to untreated control (DMSO control). (D) The cell lysates were prepared from LNCaP cells treated with BR-DIM for 24, 48 and 72 h. BR-DIM treatment showed repressed EZH2 expression at 48 and 72 h. (*, p<0.05; **, p<0.01).
(TIF)
