# Supplementary figures and images for: Loss of Let-7 Up-Regulates EZH2 in Prostate Cancer Consistent with the Acquisition of Cancer Stem Cell Signatures That Are Attenuated by BR-DIM
Source: PLoS One. 2012 Mar 19;7(3):e33729. doi: 10.1371/journal.pone.0033729 (PMC3307758; doi:10.1371/journal.pone.0033729)

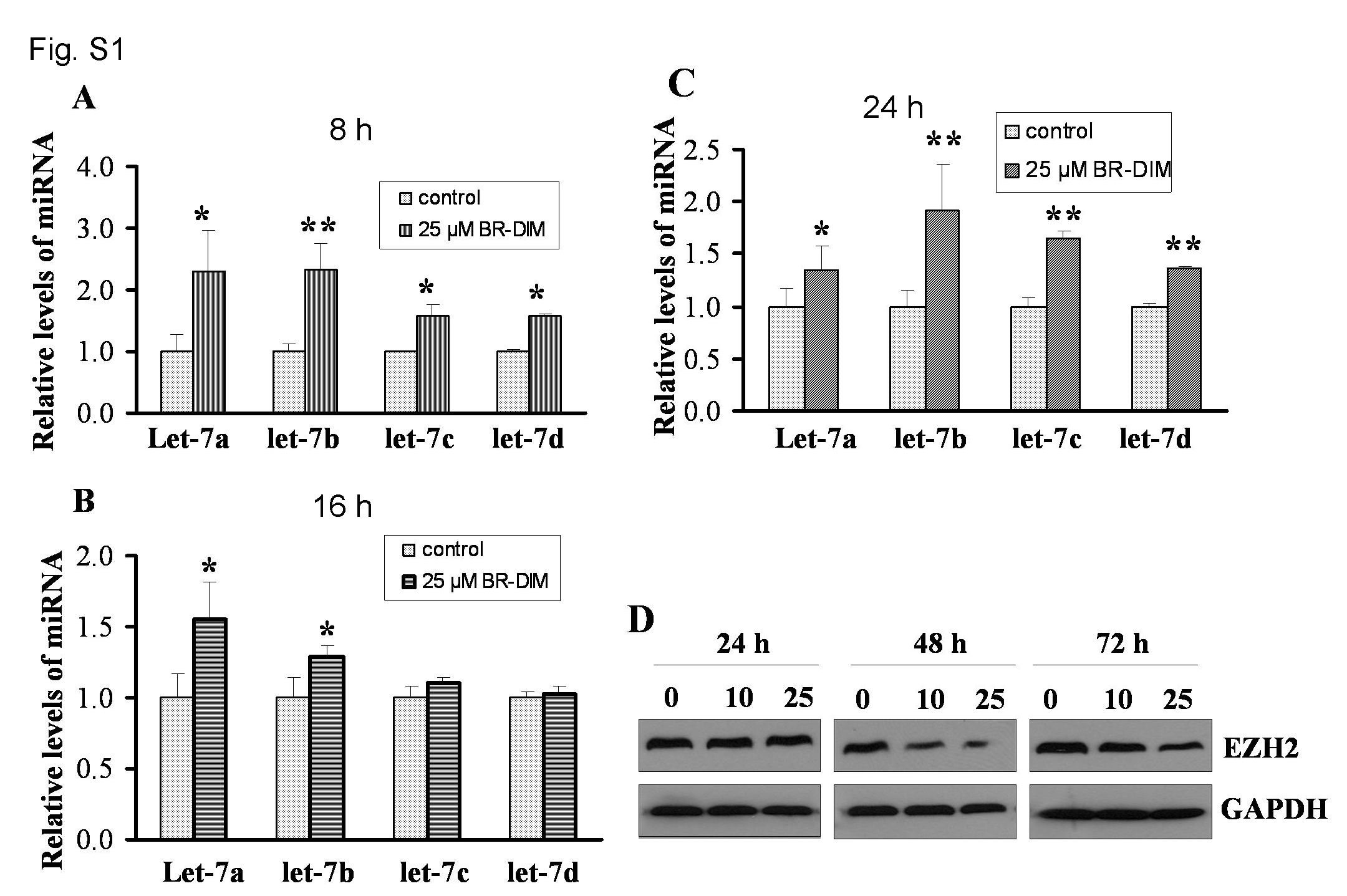

Supplement: Figure S1 — BR-DIM treatment upregulated let-7 expression and consequently reduced EZH2 expression in LNCaP cells at different time points. (A–C) Total RNA was isolated from LNCaP cells treated with 25 µM BR-DIM for 8, 16 and 24 h, and the results from real time RT-PCR were shown to document the expression of let-7, which was increased following BR-DIM treatment compared to untreated control (DMSO control). (D) The cell lysates were prepared from LNCaP cells treated with BR-DIM for 24, 48 and 72 h. BR-DIM treatment showed repressed EZH2 expression at 48 and 72 h. (*, p<0.05; **, p<0.01). (TIF) [file pone.0033729.s001.tif]
